# Exploring the prognostic significance and therapeutic potential of SUCLG2 in prostate cancer

**DOI:** 10.3389/fgene.2025.1592779

**Published:** 2025-07-17

**Authors:** Bao Hua, Qing Yang, Shangqing Song, Wenfeng Li, Bin Xu, Yufei Gu

**Affiliations:** Department of Urology, School of Medicine, Shanghai Ninth People’s Hospital, Shanghai Jiao Tong University, Shanghai, China

**Keywords:** prostate cancer, SUCLG2, lipid metabolism, single-cell RNA sequencing, personalized therapy

## Abstract

**Background:**

Prostate cancer (PCa), a highly heterogeneous cancer with a complex molecular pathogenesis, is a leading cause of cancer-related mortality among men globally. The present study presents a lipid metabolism-based risk model for PCa and explores the role of succinyl-CoA ligase GDP-forming subunit beta (SUCLG2), a potential marker and therapeutic target in PCa involved in lipid metabolism and cancer progression, from the perspective of developing effective diagnostic and therapeutic strategies.

**Methods:**

High-throughput RNA sequencing and single-cell RNA sequencing were used to investigate the expression and functional relevance of SUCLG2 in PCa. We analyzed 497 PCa samples from The Cancer Genome Atlas and conducted a comprehensive bioinformatics analysis, including univariate Cox proportional hazards regression, least absolute shrinkage and selection operator regression, and gene set enrichment analysis. Furthermore, quantitative real-time polymerase chain reaction and immunofluorescence assays were performed to validate SUCLG2 expression in clinical samples and the prostate carcinoma epithelial cell line 22Rv1.

**Results:**

Our findings revealed that lipid metabolism-related genes, including SUCLG2, have significant prognostic value, based on a 16-gene risk model constructed that accurately predicted PCa prognosis. In particular, SUCLG2 was significantly enriched in luminal and basal/intermediate cell subsets, highlighting its potential role in tumor progression and therapy resistance. Drug sensitivity analysis indicated that SUCLG2 expression is correlated with the efficacy of several chemotherapeutic agents, based on which strategies for personalized therapy in PCa treatment could be devised.

**Conclusion:**

SUCLG2 plays a pivotal role in the metabolic reprogramming of PCa, thus offering new insights into its progression and potential therapeutic targets. Our study underscores the importance of metabolic pathways in PCa pathogenesis and paves the way for the development of targeted therapies, thus contributing to personalized medicine in PCa management.

## Introduction

Prostate cancer (PCa) remains one of the most prevalent malignancies affecting men worldwide, posing significant challenges in terms of diagnosis, treatment, and management (Wang et al., 2018). The disease exhibits a highly heterogeneous clinical course that is underscored by a complex pathogenesis involving a wide array of molecular alterations and a diverse tumor microenvironment (Chen et al., 2021; Sekhoacha et al., 2022). Recent advancements in high-throughput sequencing technologies, particularly RNA sequencing (RNA-seq) and single-cell RNA sequencing (scRNA-seq), have opened new avenues for exploring the molecular landscape of PCa at a hitherto unprecedented level of depth. Further, genomics and transcriptomics studies have unveiled various subtypes of PCa characterized by distinct mutations and aberrant transcriptional profiles, shedding light on the molecular diversity underpinning the disease (Chen et al., 2019; You et al., 2016; Zhao et al., 2017).

scRNA-seq technology allows for the simultaneous assessment of thousands of cells within a sample, thus revealing the extent of heterogeneity among tumor cells (Azizi et al., 2018; Tirosh et al., 2016). This technology has highlighted the pivotal roles of luminal and basal/intermediate cells in the development and progression of PCa. Luminal cells, often linked to the differentiated state of the prostate gland, are implicated in the majority of PCa cases and are pivotal in the disease’s androgen-driven progression (You et al., 2016; Zhao et al., 2017). Conversely, basal/intermediate cells, with their stem-cell-like properties and capacity for self-renewal, are thought to contribute to tumor initiation, recurrence, and resistance to therapy, emphasizing the complexity of effectively targeting PCa (Parson et al., 2001).

With regard to the molecular mechanisms, similar to other cancers, PCa has been linked with metabolic dysregulation, and several metabolism-related markers have been associated with tumor growth, progression, and therapy resistance (Yang et al., 2024). Among the known markers, succinyl-coenzyme A (CoA) ligase GDP-forming subunit beta (SUCLG2), a significant player in the lipid metabolism pathway, has emerged as a potential prognostic marker and therapeutic target in PCa (Lin et al., 2020; Hu et al., 2023). SUCLG2 has been implicated in several key metabolic processes critical to cancer metabolism, including the tricarboxylic acid (TCA) cycle, and represents a link between altered metabolic pathways and tumor biology (Wu et al., 2020). Based on these previous findings, the current study aims to explore in more depth the expression and functional relevance of SUCLG2 within the PCa milieu and, thereby, shed light on the molecular mechanisms by which lipid metabolism-related genes influence PCa progression and identify potential avenues for targeted therapy.

Based on our molecular studies, we have constructed a risk prediction model comprising 16 genes related to lipid metabolism in PCa that shows high accuracy for identifying patients at risk of poor survival. This model could help guide the development of more effective, targeted treatment strategies that can improve patient outcomes in this complex disease landscape.

## Materials and methods

### Clinical samples

A total of 10 matched pairs of tumor and adjacent normal tissue samples were procured from patients with PCa who underwent surgery in 2021 at the Shanghai Ninth People’s Hospital, Shanghai Jiao Tong University School of Medicine. Prior to sample collection, written informed consent was obtained from all the participating patients. The research protocol received the approval of the Institutional Ethical Review Board of the Shanghai Ninth People’s Hospital, Shanghai Jiao Tong University School of Medicine, and was performed in compliance with the relevant ethical guidelines (approval no. SH9H-2021-A26-1).

### Cell culture

The human prostate carcinoma epithelial cell line 22Rv1 was obtained from the cell bank of Fudan University. Cells were cultured in Dulbecco modified Eagle medium (Gibco, Grand Island, NY, United States) supplemented with 10% heat-inactivated fetal bovine serum (Gibco, Grand Island, NY, United States), 100 U/mL penicillin, and 100 μg/mL streptomycin (Gibco, Grand Island, NY, United States), in a humidified atmosphere containing 5% CO_2_ and 95% air at 37°C.

### Data collection

RNA sequencing data and clinical information for PCa samples were obtained from The Cancer Genome Atlas (TCGA) database (https://portal.gdc.cancer.gov) (Xu et al., 2023). A total of 497 prostate cancer samples were selected for further analysis. Samples with incomplete clinical information and normal tissue samples were excluded. A comprehensive list of 859 lipid metabolism-related genes was curated from “Reactome metabolism of lipids and lipoproteins,” “Reactome phospholipid metabolism,” “Hallmark fatty acid metabolism,” and “KEGG glycerophospholipid metabolism” in the Molecular Signatures Database (MSigDB: https://www.gsea-msigdb.org/gsea/msigdb/index.jsp). This gene list served as the foundation for subsequent analyses. Immunohistochemical data for SUCLG2 expression in tissues were obtained from the Human Protein Atlas (https://www.proteinatlas.org).

### Data preprocessing

To identify lipid metabolism genes with prognostic significance, univariate Cox proportional hazards regression analysis was performed on RNA-seq data, with a focus on the curated list of lipid metabolism genes. Genes with a p-value less than 0.05 were considered to have significant prognostic value. Least absolute shrinkage and selection operator (LASSO) regression analysis was employed to construct a risk model based on the prognostically significant genes identified with the R package “glmnet” (Xu et al., 2021). This method was chosen for its efficacy in handling high-dimensional data and its ability to enhance model predictability by imposing a penalty on the absolute size of the coefficients. The following formula was used for calculating risk scores based on the expression levels of lipid metabolism-related genes: Risk score = ∑ni = ∑Coefi × xi, where xi stands for the expression level of gene i, and Coefi, for the regression coefficient. Based on the median risk score, the patients were divided into high- and low-risk groups.

Kaplan-Meier survival analysis was employed to evaluate the disparities in overall survival (OS), disease-free survival (DFS), and progression free survival (PFS) between the high- and low-risk groups. The precision of the risk model was gauged using receiver operating characteristic (ROC) curve analysis. Additionally, the expression profiles of the genes associated with lipid metabolism risk were depicted through heatmaps, utilizing the “pheatmap” package in R for visualization.

### Single-cell RNA sequencing analysis

Single-cell RNA sequencing data from the included patients were downloaded from the GSE141445 dataset. The initial phase involved rigorous quality control measures to ensure the reliability of subsequent analyses. Cells were selected based on a set of predefined criteria aimed at excluding low-quality or ambiguous cells, thus refining the dataset for more accurate clustering and analysis. The criteria for inclusion were as follows: (1) expression of 200 to 5,218 distinct genes that can be used to capture a comprehensive, yet precise, transcriptomic profile; (2) mitochondrial gene expression under 20% to mitigate the influence of cellular stress or apoptosis, which might otherwise skew the findings; (3) a minimum transcript count threshold of 1,000 to filter out cells potentially compromised by inadequate RNA content, probably due to technical discrepancies.

Following quality control based on the above criteria, the “Seurat” package in R was utilized for data normalization, variance stabilization, and scaling. The dimensionality reduction techniques t-distributed Stochastic Neighbor Embedding (tSNE) and Uniform Manifold Approximation and Projection (UMAP) were employed to visualize the cellular landscapes and the distribution of the identified cell types, and thereby, provide insights into the complex architecture of PCa tissues at the single-cell level. A list of marker genes for different cell types was collected to annotate the cell clusters (Chen et al., 2021).

### Functional enrichment analysis

Gene Ontology (GO) and Gene Set Enrichment Analysis (GSEA) were conducted using the “clusterProfiler” R package to identify the biological functions and pathways linked with the identified genes, with a focus on SUCLG2. This analysis aimed to illuminate the potential mechanisms by which these genes influence the progression of PCa.

### Quantitative real-time polymerase chain reaction

For RNA extraction and qPCR analysis, total RNA was isolated from 10 matched pairs of tumor (PCa) and adjacent normal tissues utilizing the TRIzol reagent (Invitrogen, Carlsbad, CA, United States), with strict adherence to the manufacturer’s guidelines. After extraction, the concentration of the retrieved RNA was determined, and this was followed by the synthesis of cDNA with the RevertAid First Strand cDNA Synthesis Kit (Takara Bio, Shiga, Japan), as per the kit’s specifications. RNA was quantified in accordance with the protocol of the TRIzol reagent obtained from Invitrogen, and the extracted RNA was reverse-transcribed into cDNA using a dedicated reverse transcription kit. PCR amplification and detection were carried out in a 20 μL reaction volume utilizing the BIO-RAD fluorescent real-time PCR system. The sequences of the SUCLG2 primers were as follows:

Forward: 5ʹ-TGT​TGG​TGG​TGG​TGC​TAC​AG-3ʹ,

Reverse: 5ʹ-TCG​TGT​ACC​TTG​TAA​CCG​TAC​C-3ʹ.

### Immunofluorescence assay

A total of 1,000 cells of the prostate carcinoma epithelial cell line 22Rv1 were plated per well in a 24-well plate containing cell slides (NEST 801 010) and incubated for 24 h. Subsequently, the slides were rinsed thrice with phosphate-buffered saline (PBS) for 5 min each, and this was followed by fixation in 4% paraformaldehyde for 30 min. After fixation, the cells were washed three more times with PBS and then blocked with a solution containing 3% BSA and 0.2% Triton X-100 in PBS for 1 h at ambient temperature. The cells were then incubated overnight with primary rabbit anti-SUCLG2 antibodies (1:250, ab187996) and rinsed five times over a 15-min span using a wash buffer (containing 0.2% BSA and 0.05% Triton X-100 in PBS), after which they were incubated for 1 h with fluorescent-tagged secondary donkey anti-rabbit IgG (1:300, ab150068). This was followed with three rinses with the wash buffer, and then DAPI (0100–20, Southern Biotech) was applied for nuclear staining. Imaging was subsequently performed using a laser confocal microscope (ZEISS).

### Drug sensitivity analysis

Drug sensitivity analysis was performed to explore the correlation between SUCLG2 expression and responsiveness to various chemotherapeutic agents, by utilizing the comprehensive drug sensitivity datasets available in the CellMiner database (https://discover.nci.nih.gov/cellminer/home.do). This segment of the study concentrated on identifying medications for which the half-maximal inhibitory concentration (IC50) values demonstrated a significant association with the expression levels of SUCLG2.

Pearson correlation coefficients were calculated to assess the strength and direction of the relationship between SUCLG2 expression and the IC50 values for each drug. Drugs exhibiting a statistically significant correlation (p < 0.05) were highlighted as potential candidates for targeted therapy of PCa based on altered SUCLG2 expression.

### Statistical analysis

SPSS version 23.0 (SPSS Inc., Chicago, IL, United States) and R software for Windows version R-4.3.3 (The R Foundation for Statistical Computing, Vienna, Austria) were used for data analysis. P values <0.05 were considered to indicate statistical significance.

## Results

### Construction and Validation of a risk model comprising lipid metabolism genes

**FIGURE 1:**
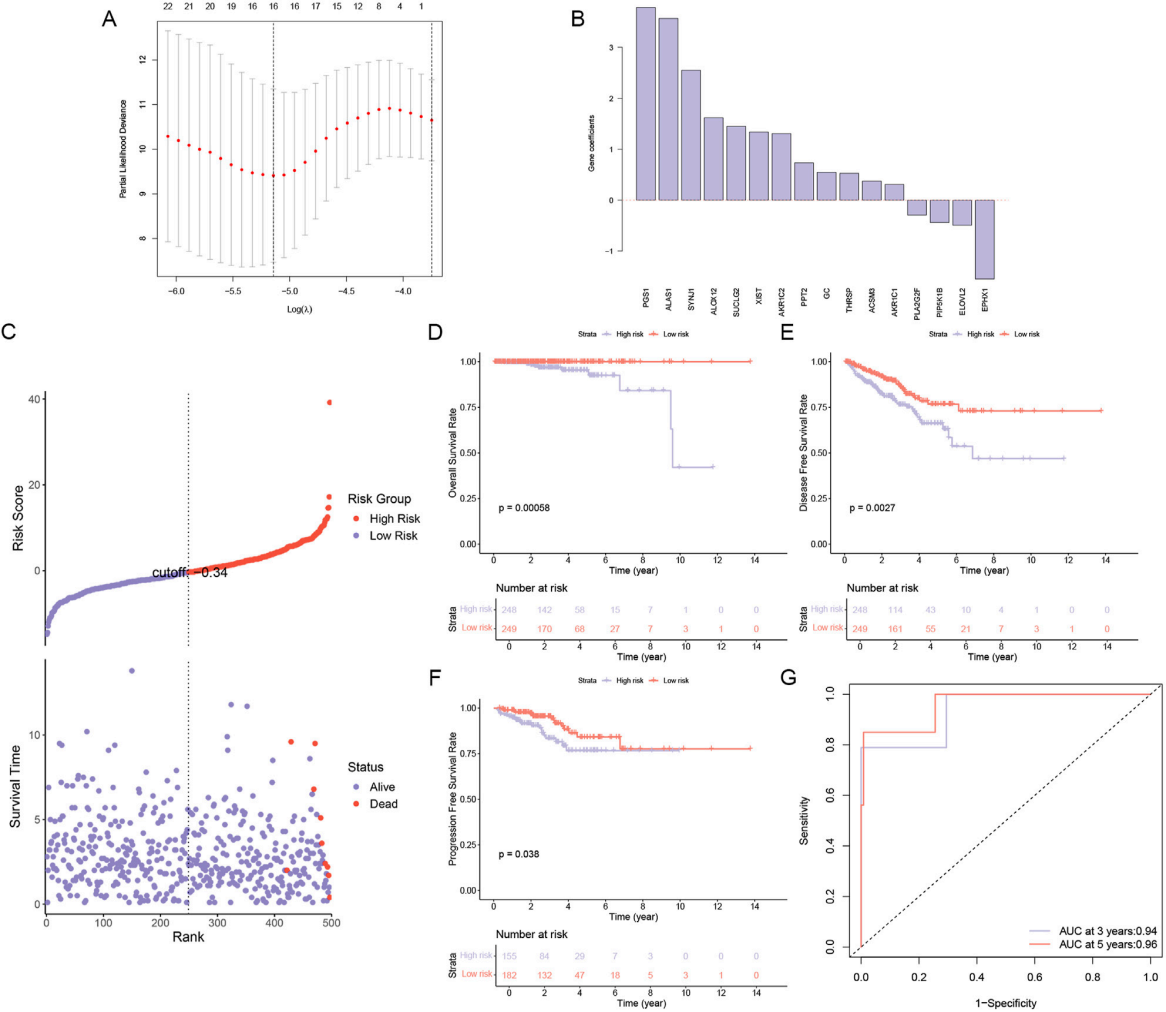
Construction and Validation of a Lipid Metabolism Gene Risk Model. **(A)** A LASSO regression model was constructed to identify 16 lipid metabolism-related genes associated with high risk in PCa. **(B)** Gene coefficients of genes related to the risk of PCa progression. **(C)** Risk score and survival time in high- and low-risk groups, **(D)** Overall survival analysis in high- and low-risk groups. **(E)** Disease-free survival analysis in high- and low-risk groups. **(F)** Progression-free survival analysis in high- and low-risk groups. **(G)** AUC curve for prediction accuracy.

In our quest to unravel the complex role of lipid metabolism in PCa, we embarked on a rigorous analysis of RNA-seq data from 497 PCa samples obtained from the TCGA database. By integrating these samples with a curated list of 859 lipid metabolism genes from the MSigDB database, we conducted a comprehensive univariate Cox regression analysis and identified 41 genes with significant prognostic value (p < 0.05). Leveraging the predictive power of these genes, we constructed a robust LASSO model that culminated in a 16-gene risk model that could accurately predict the prognosis of PCa (Figure 1A). As shown in Figure 1B, the gene coefficients represent the weight of the influence of each gene in the PCa sample. Based on the model, the cohort was divided into high-risk and low-risk groups with surgical precision, and a sharp contrast in mortality was observed between the two groups (Figure 1C). Survival analysis indicated that significantly better OS, DFS, and PFS were observed in the low-risk group (Figures 1D–F). Further, the ROC curve (Figure 1G) validated the accuracy of the model.

### Clinical correlation and expression heatmap of the risk model comprising 16 lipid metabolism-related genes

**FIGURE 2:**
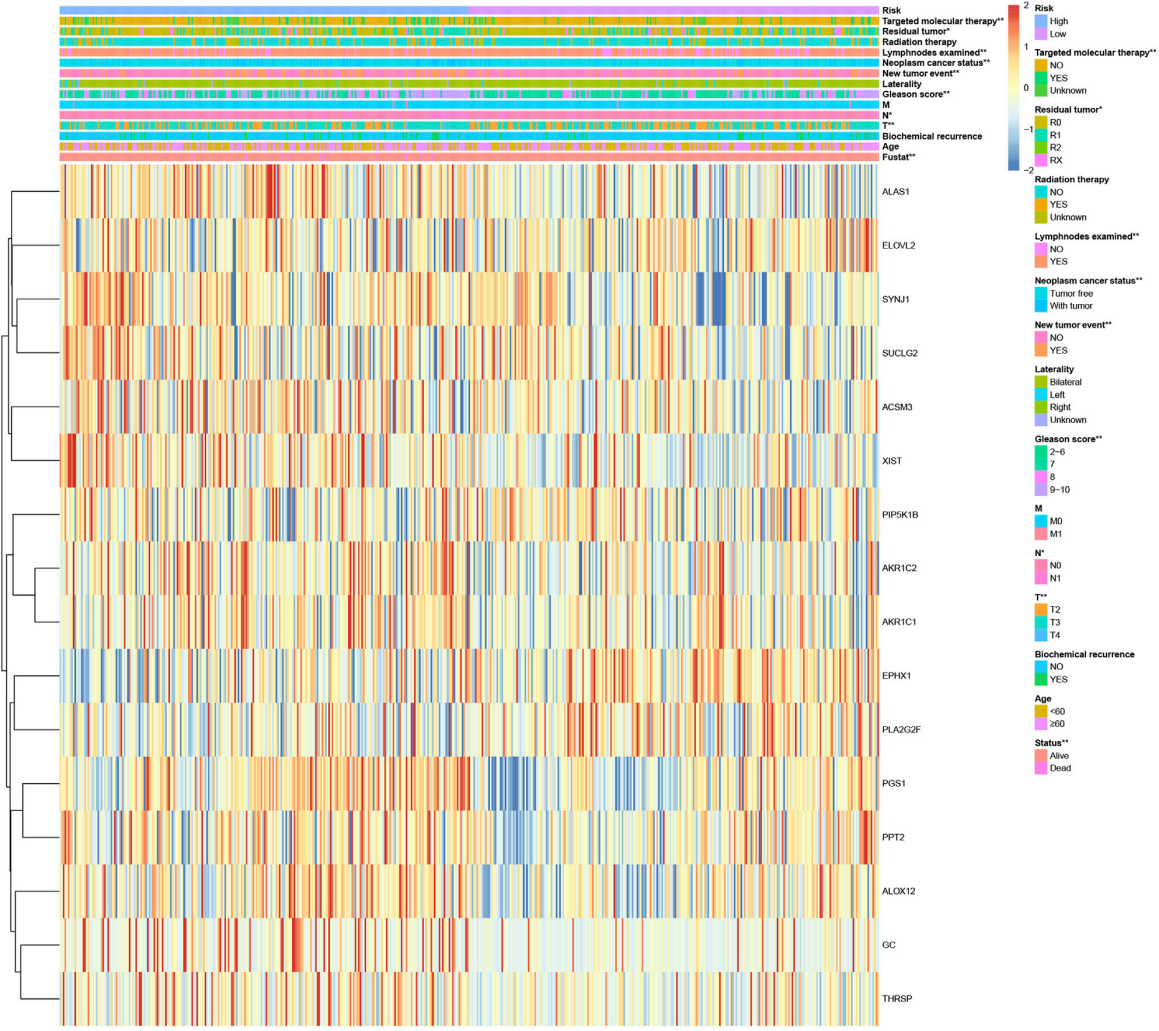
Clinical correlation and expression heatmap of the risk model comprising 16 lipid metabolism-related genes. *p < 0.05 and **p < 0.01.

We analyzed the correlation between the expression of the 16 lipid metabolism-related genes and clinicopathological characteristics (Figure 2), and the results showed that the expression of these 16 lipid metabolism-related genes was significantly correlated with clinical features such as T stage, N stage, Gleason score, new tumor event, neoplastic cancer status, number of lymph nodes examined, residual tumor, targeted molecular therapy, and survival status. In addition, SUCLG2, PPT2, PGS1, ALAS1, SYNJ1, ALOX12, ACSM3, THRSP, AKR1C1, AKR1C2, XIST, and GC were mainly expressed in the high-risk group. This implies that these 12 genes may be closely related to the poor prognosis of PCa.

### Cellular heterogeneity based on single-cell RNA sequencing

**FIGURE 3:**
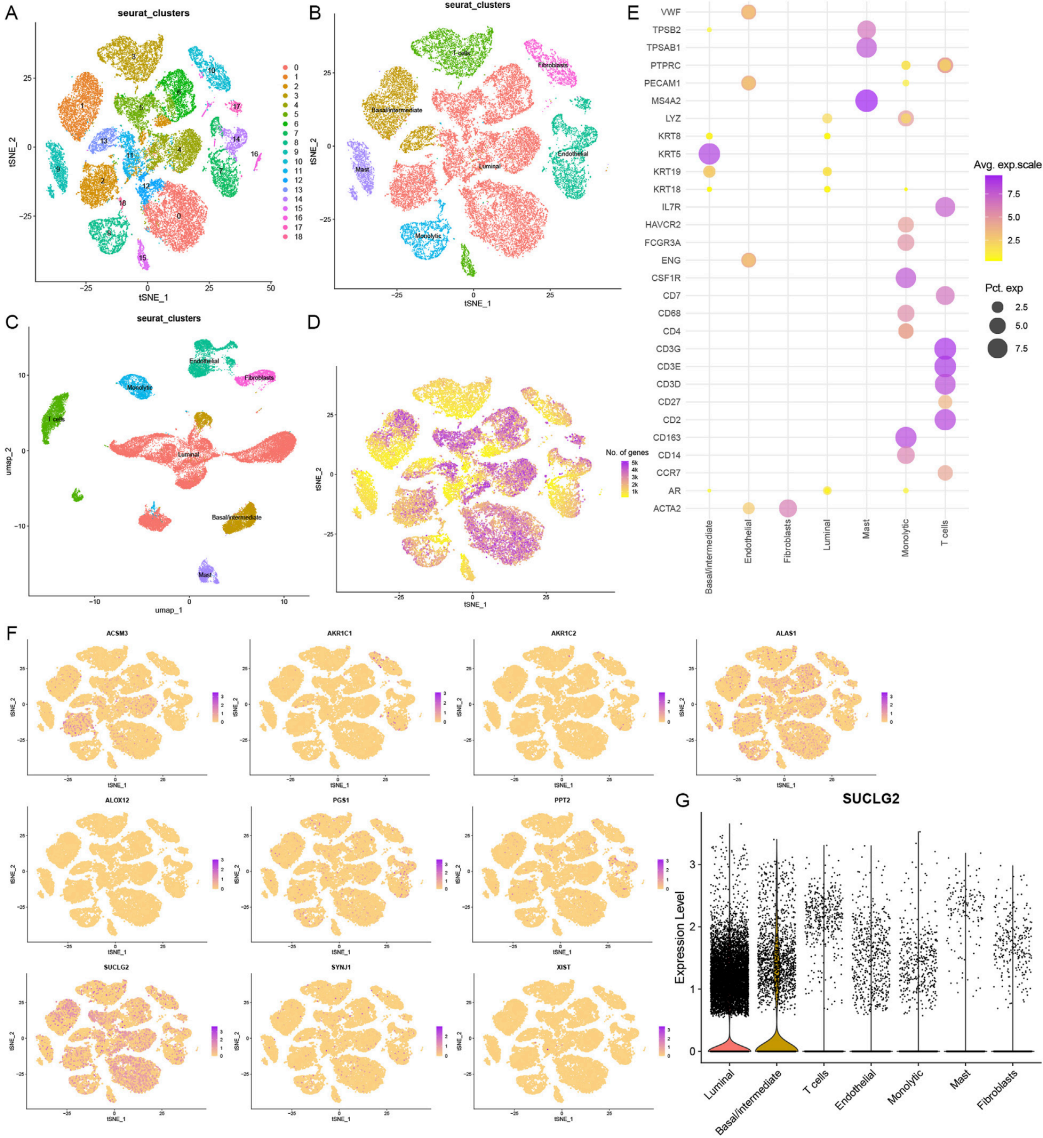
Cellular Heterogeneity based on Single-Cell RNA Sequencing. **(A)** A t-distributed stochastic neighbor embedding (tSNE) view of 35,405 single cells, color-coded by assigned cell clusters. **(B)** tSNE view of 7 cell subsets. **(C)** Uniform Manifold Approximation and Projection (UMAP) plot of 7 cell clusters. **(D)** tSNE view of all cells, color-coded by the number of genes detected in each cell. **(E)** Expression of marker genes for each cell type, where dot size and color represent the percentage of marker gene expression (pct. exp) and the averaged scaled expression (avg. exp. scale) value, respectively. **(F)** tSNE view of the expression of 10 lipid metabolism-related risk genes in 7 cell subsets. **(G)** Violin plot of SUCLG2 expression in 7 cell subsets.

To further investigate the cellular landscape of PCa, we employed single-cell RNA sequencing to dissect the heterogeneity of cell subsets. By analyzing 13 samples (12 sample of primary PCa and 1 sample of lymphocyte node metastasis) from 12 patients in the GSE141445 dataset, we screened and obtained 35,405 cells that were classified under 19 different cell clusters (Figure 3A). We used reported cell markers for detailed annotation of cell identity (Chen et al., 2021). In addition, tSNE and UMAP plots were drawn to observe the local characteristics and overall distribution of cell subsets (Figures 3B,C). Finally, we were able to confirm the presence and distribution of seven cell subsets, namely, luminal cells, basal/intermediate cells, T cells, endothelial cells, fibroblasts, monocytic cells, and mast cells.

Basal/intermediate and luminal cell subsets were found to express many of the identified lipid metabolism genes (Figure 3D). Except for GC and THRSP, the other high-risk lipid metabolism genes were broadly expressed across different cell subsets (Figure 3F). In particular, SUCLG2 was significantly enriched in the basal/intermediate and luminal cell subsets. This suggests that SUCLG2 may contribute to PCa progression via its activity in these two key epithelial cell populations (Figure 3G).

### Role of SUCLG2 in PCa based on functional enrichment analysis

**FIGURE 4:**
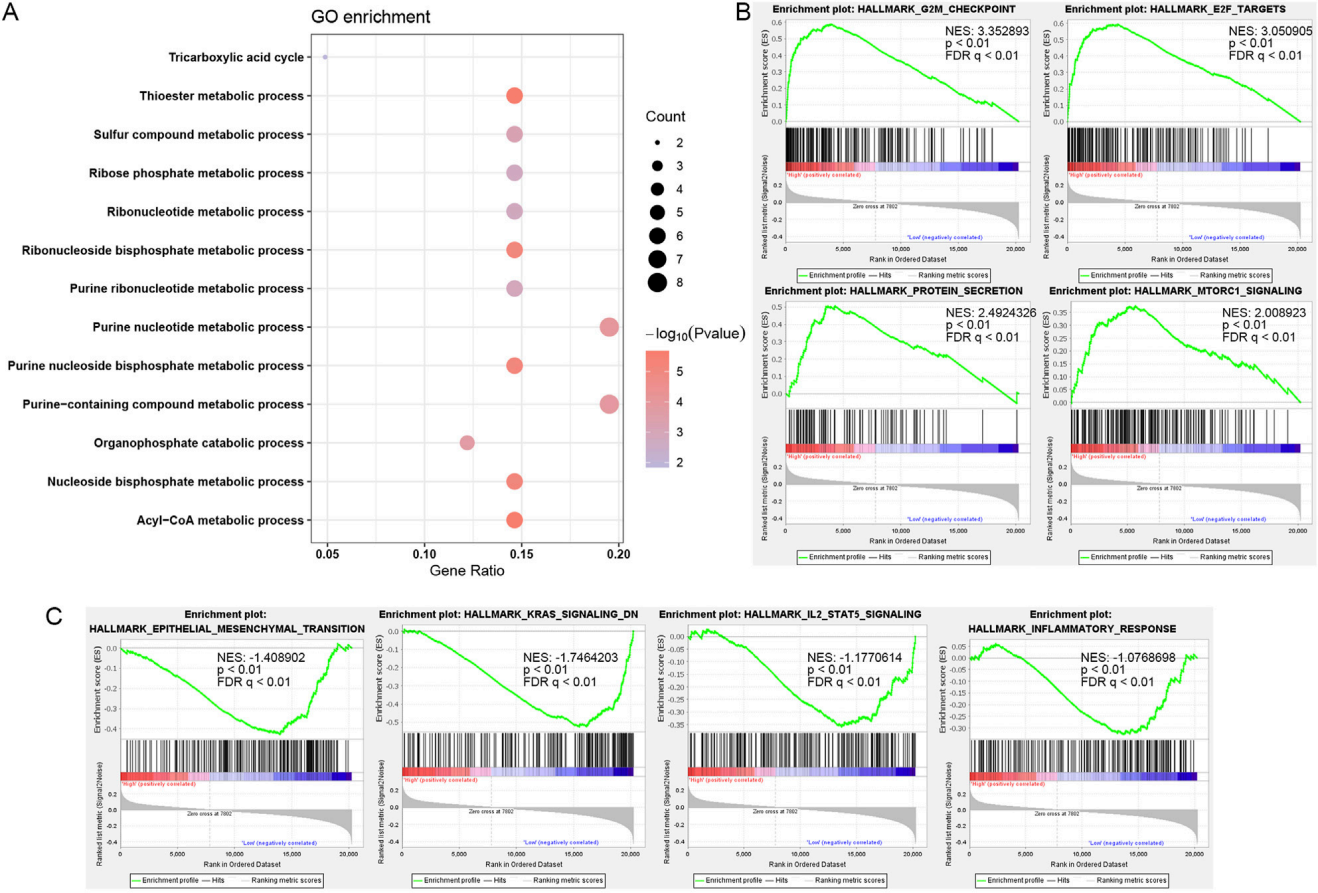
Role of SUCLG2 in PCa based on Functional Enrichment Analysis. **(A)** Gene Ontology (GO) enrichment for 41 lipid metabolism genes with significant prognostic value. **(B)** Gene Set Enrichment Analysis (GSEA) results related to high SUCLG2 expression. **(C)** GSEA results related to low expression of SUCLG2. NES, normalized enrichment score; FDR q, false discovery rate q value.

To decipher the functional roles of SUCLG2 in PCa, we performed a comprehensive GO analysis on the 41 genes identified from the univariate Cox regression analysis and found that SUCLG2 played a significant role in multiple metabolic processes. This analysis pointed out SUCLG2’s involvement in crucial metabolic pathways, including the acyl-CoA metabolic process, the thioester metabolic process, and purine metabolism, and the results were indicative of its central role in the metabolic reprogramming of PCa cells (Figure 4A).

Subsequent GSEA further delineated the dichotomy in the biological pathways enriched among samples with high versus low SUCLG2 expression. That is, samples with elevated SUCLG2 levels were predominantly associated with cell cycle checkpoints and protein secretion pathways that were indicative of the potential mechanisms by which SUCLG2 may contribute to tumor aggressiveness (Figure 4B). Conversely, samples with lower SUCLG2 expression were enriched in pathways related to epithelial–mesenchymal transition and inflammatory responses, highlighting the multifaceted roles of SUCLG2 in tumor biology (Figure 4C).

The dual behavior of SUCLG2, associated with proliferative pathways at high expression and inflammatory/EMT pathways at low expression, may indicate a stage- or microenvironment-specific role. In early-stage tumors, SUCLG2 may promote biosynthetic and proliferative programs, while in inflamed or therapy-resistant settings, reduced SUCLG2 may shift the tumor state toward EMT and immune evasion.

### Validation of SUCLG2 expression in PCa

**FIGURE 5:**
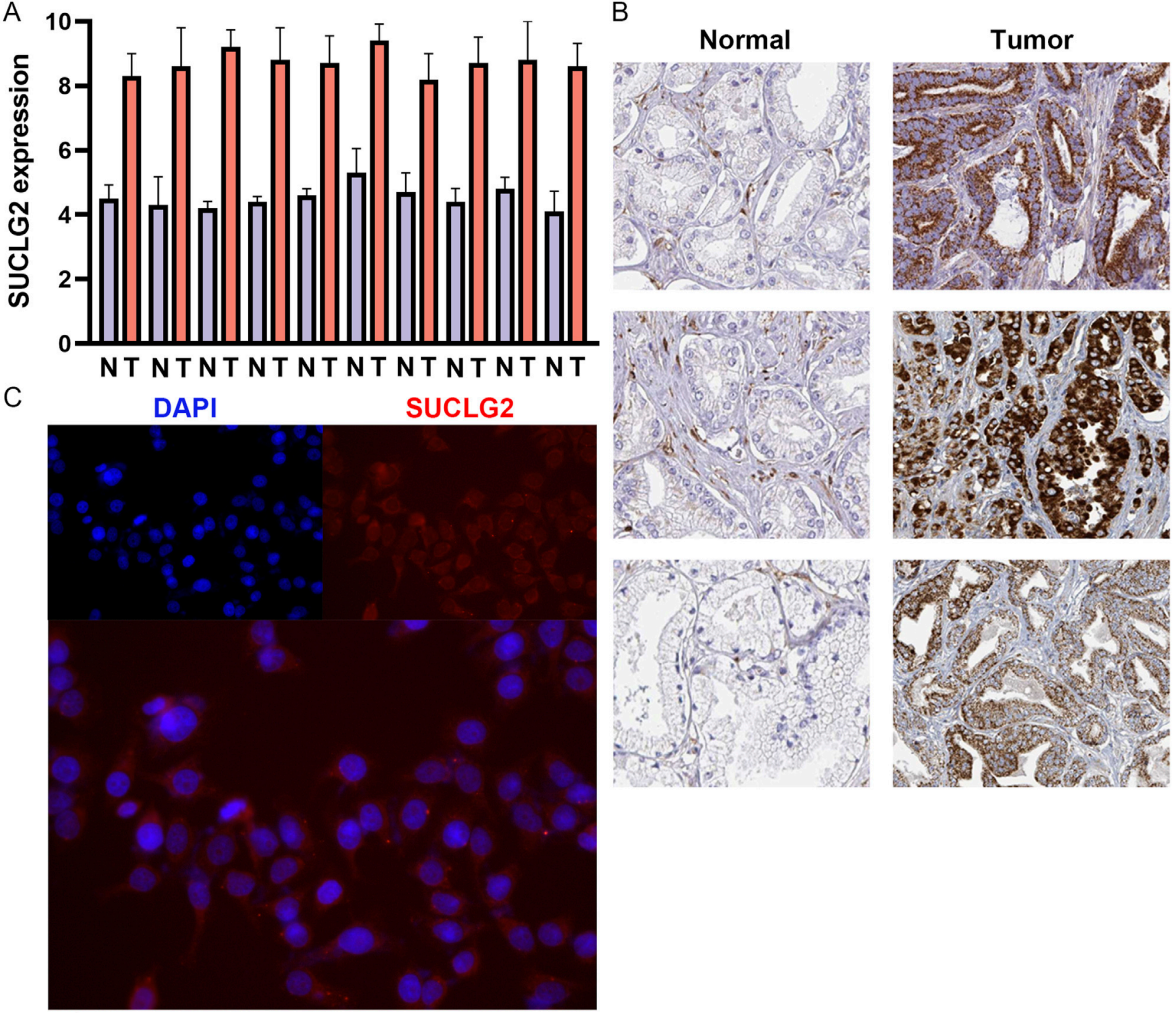
Validation of SUCLG2 Expression in PCa. **(A)** Quantitative real-time PCR analysis of 10 matched pairs of tumor and adjacent normal tissues. **(B)** Immunohistochemical data from the Human Protein Atlas dataset. **(C)** Immunofluorescence assay of SUCLG2 expression in the prostate carcinoma epithelial cell line 22Rv1.

To validate the differential expression of SUCLG2 in PCa, we conducted qPCR analysis to compare the mRNA expression of SUCLG2 in 10 pairs of tumor tissues and adjacent normal tissues. The results unequivocally confirmed higher expression levels of SUCLG2 in tumor tissues, reinforcing its relevance in PCa pathology (Figure 5A). Immunohistochemical data from The Human Protein Atlas further corroborated the elevated expression of SUCLG2 in PCa tissues, particularly within glandular epithelial cells, thus providing a histological perspective on its role in tumor biology (Figure 5B).

SUCLG2 is mainly involved in the transcription and translation of enzymes involved in the TCA cycle and energy metabolism in mitochondria, and accordingly, these enzymes are mainly expressed in the mitochondria of cells (Hu et al., 2023). Accordingly, immunofluorescence analysis of the PCa epithelial cell line 22Rv1 revealed the cytoplasmic localization of SUCLG2 and provided insights into its cellular function and potential mechanism of action in tumor progression (Figure 5C).

### Drug sensitivity analysis

From the viewpoint of precisely tailoring PCa treatment to the needs of individual patients, our study ventured into the realm of pharmacogenomics to identify potential drugs that could serve as targeted therapies based on SUCLG2 expression levels. By delving into the comprehensive drug sensitivity data available in the Cellminer database, we meticulously analyzed and identified nine drugs that exhibited significant correlation with SUCLG2 expression levels in PCa cells.

**FIGURE 6:**
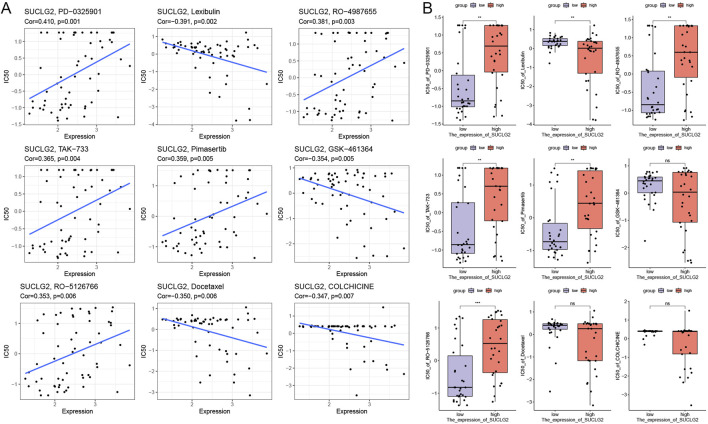
Results of Drug Sensitivity Analysis. **(A)** Correlation between gene expression and drug sensitivity for the top 9 drugs used for PCa treatment. **(B)** Relationship between gene expression and drug sensitivity in groups with high and low SUCLG2 expression. Cor, correlation coefficient; NS, not significant; **p < 0.01; ***p < 0.001.

Our analysis brought to light that the IC50 values of PD-0325901, RO-4987655, TAK-733, pimasertib, and RO-5126766 are positively correlated with SUCLG2 expression; thus, these drugs may exhibit increased efficacy in tumors with high SUCLG2 expression (Figure 6A) and may represent viable options for a more targeted and effective treatment strategy in patients with elevated levels of SUCLG2. Conversely, colchicine, docetaxel, GSK-461364, and lexibulin showed a negative correlation with SUCLG2 expression. Intriguingly, while colchicine, docetaxel, and GSK-461364 did not demonstrate significant differences in IC50 values between the high and low SUCLG2 expression groups, lexibulin displayed significantly higher IC50 values in the low expression group than in the high expression group (Figure 6B). This subtle relationship suggests that lexibulin, in particular, might be more suitable for patients with lower SUCLG2 levels and present a therapeutic avenue that could potentially protect patients from unnecessary toxicity while targeting the tumor more effectively.

## Discussion

The present study intricately dissects the multifaceted landscape of PCa, delving deep into the lipid metabolism-related molecular mechanisms involved in its progression, specifically in terms of the role of SUCLG2. Through a risk model comprising 16 significant lipid metabolism genes as risk factors and comprehensive genomic and transcriptomic analyses coupled with functional assays, this research illuminates the prognostic significance of lipid metabolism genes in PCa and underscores SUCLG2’s potential as a therapeutic target. Overall, our findings resonate with, and extend upon, the existing literature, affirming the complexity and heterogeneity of PCa pathogenesis and progression.

The novel 16 lipid metabolism-related gene risk model presented here holds the promise of transforming patient management by stratifying risk with hitherto unprecedented precision. The 16 lipid metabolism-related genes included in this model may be associated with the abnormal clinicopathological features of PCa patients, and in particular, 12 of them are more likely to lead to poor prognosis of PCa patients. This risk model not only highlights the prognostic potential of these genes in PCa but is also in alignment with previous studies advocating for the significance of metabolic pathways in cancer biology. Beyond prognostic prediction, the lipid metabolism-based risk model may also offer utility in identifying patients with aggressive disease features or in predicting treatment response, such as metabolic therapy or combination regimens. Future studies are warranted to validate the model’s application in broader clinical contexts. In particular, studies have shown that alterations in lipid metabolism contribute to the development and progression of various cancers, including PCa, by supporting membrane biosynthesis, energy production, and signaling molecule generation (Martin-Perez et al., 2022; Currie et al., 2013; Bian et al., 2021). Further, it supports previous notions by demonstrating that dysregulated lipid metabolism is a hallmark of tumor growth and survival (Hopkins et al., 2018).

The pivotal role of SUCLG2 in PCa that emerged from our study is notable, positioning this gene as a key contributor to the metabolic reprogramming in PCa cells. Our findings are supported by Faubert et al. and Sun et al., who identified metabolic reprogramming as a critical factor in cancer progression, underscoring the importance of targeting metabolic pathways for cancer therapy (Faubert et al., 2020; Sun et al., 2022). The association between high SUCLG2 expression and aggressive PCa features, such as advanced tumor stage and poor prognosis, dovetails with recent discoveries in metabolic oncology. For instance, Wu et al. and Shen et al. reported that alterations in the TCA cycle enzymes, which SUCLG2 also contributes to, are indicative of a more malignant cancer phenotype, thus corroborating our observations (Wu et al., 2020; Shen et al., 2023).

The cellular heterogeneity within PCa significantly contributes to its diagnostic and therapeutic challenges. Particularly, the distinct roles of luminal and basal/intermediate cells in the disease’s pathogenesis and progression have garnered considerable attention. Our study’s findings, which emphasize the differential expression of SUCLG2 across these cellular subsets, offer novel insights into the metabolic reprogramming in PCa and its potential exploitation for therapeutic advances. Luminal cells, characterized by their expression of cytokeratin 8/18, are pivotal in the prostate gland’s differentiated state and are implicated in the majority of PCa cases (Zhang et al., 2018; Dong et al., 2020). These cells are central to the androgen-driven progression of PCa, reflecting the dominant role of androgen receptors in the biology of these cells and, by extension, in the pathophysiology of PCa (Aurilio et al., 2020). Conversely, basal/intermediate cells, marked by cytokeratin 5/14 expression, possess stem-cell-like properties, including the capacity for self-renewal and differentiation. These cells are hypothesized to contribute to tumor initiation, recurrence, and therapy resistance (Le Magnen et al., 2018). The distinct nature of these cellular compartments underscores the complexity of PCa and highlights the need for targeted therapeutic strategies that consider this cellular diversity.

Our analysis revealed that SUCLG2 is significantly enriched in both luminal and basal/intermediate cell subsets. This finding is critical, considering the enzyme’s role in the TCA cycle and, by extension, in the metabolic landscape of PCa cells. The association of SUCLG2 with these cellular subsets suggests a link between metabolic processes and cellular differentiation states within the tumor microenvironment. Specifically, the elevated expression of SUCLG2 in luminal cells might contribute to the androgen-driven metabolic phenotype observed in the majority of PCa cases, potentially offering a mechanistic insight into the role of lipid metabolism in androgen receptor signaling (Butle et al., 2016). Moreover, the presence of SUCLG2 in basal/intermediate cells hints at the metabolic flexibility of these cells, which may underlie their role in tumor initiation and resistance to therapy. Thus, the stem-cell-like properties of basal/intermediate cells, coupled with their metabolic reprogramming mediated by SUCLG2, could provide a survival advantage under the selective pressures imposed by therapeutic interventions (Pfeiffer and Schalken, 2010). The enrichment of SUCLG2 in both luminal and basal/intermediate cell subsets may suggest context-dependent roles in prostate cancer biology. While luminal cells are typically associated with androgen signaling and differentiated tumor states, basal/intermediate cells are known for their stem-like, therapy-resistant features. The presence of SUCLG2 in both may indicate its involvement in both proliferative and adaptive metabolic programs, contributing to tumor heterogeneity and progression.

Overall, the functional analysis not only uncovers the diverse biological processes influenced by SUCLG2 but also underscores its potential as a pivotal player in the metabolic and proliferative aspects of PCa progression.

The drug sensitivity analysis conducted in this study not only furthers our understanding of the role of SUCLG2 in PCa but also opens up new avenues for personalized therapy. By correlating SUCLG2 expression levels with the efficacy of specific chemotherapeutic agents, we identify potential targeted therapies for PCa. Interestingly, the positive correlation between SUCLG2 expression and sensitivity to MEK inhibitors suggests that SUCLG2 may modulate or reflect MAPK pathway activity. While the mechanistic link remains to be fully elucidated, our findings raise the possibility that SUCLG2 could serve as an indirect marker for responsiveness to MAPK-targeted therapies in PCa. This approach mirrors the sentiments of Shemesh et al., who advocate for personalized medicine based on the molecular profiles of tumors to enhance treatment efficacy and minimize side effects (Shemesh et al., 2021). Through our comprehensive drug sensitivity analysis, we have not only illuminated the intricate connection between SUCLG2 expression and drug response in PCa but also identified specific drugs that could be leveraged for personalized treatment approaches. Specifically, our identification of drugs such as PD-0325901, RO-4987655, TAK-733, pimasertib, and RO-5126766, whose effectiveness may be heightened in PCa with elevated SUCLG2 expression, provides a promising foundation for future clinical trials and therapeutic strategies. Overall, the findings exemplify the potential of integrating molecular characteristics into therapeutic decision-making, heralding a new era of personalized medicine in the fight against PCa.

A notable limitation of our study is the reliance on computational and *in vitro* analyses to elucidate the role of SUCLG2 in PCa progression. While these approaches have provided valuable insights into the potential mechanisms by which SUCLG2 contributes to PCa, the complexity of cancer biology necessitates further validation through *in vivo* and *ex vivo* experiments. Specifically, the mechanistic role of SUCLG2 in PCa, especially in terms of its impact on tumor growth, metastasis, and response to therapy, remains to be fully elucidated. Future studies employing animal models and patient-derived tumor xenografts are essential to validate the therapeutic potential of targeting SUCLG2 in PCa. Additionally, exploring SUCLG2’s interactions with the tumor microenvironment and its influence on cellular metabolism within the context of a living organism will offer a more comprehensive understanding of its role in cancer progression. These investigations will not only affirm the findings of our current study but also pave the way for translating these insights into clinical applications, potentially leading to more effective treatment strategies for PCa.

## Conclusion

Our study emphasizes the intricate relationship between lipid metabolism and PCa progression, with SUCLG2 emerging as a significant prognostic marker and potential therapeutic target. By shedding light on the molecular mechanisms of PCa and identifying novel therapeutic avenues, this research contributes to the ongoing quest for more effective, personalized treatment strategies for PCa. As we move forward, the integration of metabolic profiling with clinical parameters promises to refine our approach to PCa management through therapies tailored to the unique molecular landscape of each patient’s tumor.

## Data Availability

The datasets presented in this study can be found in online repositories. The names of the repository/repositories and accession number(s) can be found in the article/supplementary material.
